# Valproate-Induced Thrombocytopenia: A Case Report

**DOI:** 10.7759/cureus.65670

**Published:** 2024-07-29

**Authors:** Kamalakar Surineni, Emilee Wells, Nolan Schrader, Gilmar Costa, Maggie Ziegler

**Affiliations:** 1 Psychiatry, University of Kansas Medical Center, Wichita, USA; 2 Psychiatry and Behavioral Sciences, University of Kansas School of Medicine-Wichita, Wichita, USA

**Keywords:** blood platelets, thrombocytopenia, drug-induced thrombocytopenia, depakote, valproic acid

## Abstract

Despite its effectiveness in treating a variety of neurologic and psychiatric conditions, valproate carries many clinically significant adverse effects that are sometimes life-threatening. Due to the potentially severe nature of these adverse effects, providers must communicate these risks to patients and maintain close follow-up, especially during the first six months following drug initiation or dose increase. We present a case of a 64-year-old male with schizoaffective disorder who developed thrombocytopenia following the initiation of valproate. The patient was started on valproate for underlying mental illness, sleep disturbance, and impulsivity, with subsequent development of thrombocytopenia, which required discontinuation of valproate. Platelet levels returned to baseline within two weeks after discontinuation of valproate. This case underscores the potential risk of developing thrombocytopenia with valproate use and highlights the importance of vigilant monitoring and consideration of underlying risk factors before the initiation of therapy.

## Introduction

First approved for the treatment of epilepsy in 1967, valproate remains a mainstay therapeutic medication for a variety of seizure disorders and psychiatric conditions [1]. It is United States Food and Drug Administration (FDA) approved for complex partial seizures and absence seizures in adult and pediatric patients and as adjunctive therapy for patients with multiple seizure types. In addition, it is FDA-approved for treating acute mania, acute mania with mixed episodes, and migraine prophylaxis [2]. Despite being widely used as an off-label medicine for diabetic peripheral neuropathy, postherpetic neuralgia, and agitation, there is little evidence to support its efficacy for these indications. In fact, Abbott Pharma was fined 1.5 billion for the off-label promotion of valproate for agitation and aggression in dementia patients [3].

Though the mechanism of action is not fully understood, potential mechanisms include increasing gamma-aminobutyric acid (GABA) levels [4], blocking voltage-gated sodium channels [5], inhibiting histone deacetylases, and increasing lymphoid enhancer-binding factor 1 [6]. Common side effects include gastrointestinal symptoms and headaches that usually subside with time. Life-threatening complications, such as thrombocytopenia, hepatic failure, and pancreatitis, have also been reported, necessitating careful dosing and monitoring during its use [2]. Rarely do such complications arise that require medication discontinuation. This case report describes an incident of valproate-induced thrombocytopenia in a patient with schizoaffective disorder during an extended stay in the inpatient psychiatric unit.

Although well-tolerated, certain patient characteristics and comorbid medical conditions are thought to confer an elevated risk of fatal side effects among patients taking valproate. Clinicians must be aware of patient populations susceptible to life-threatening events before the initiation of valproate therapy. Black-box warnings for valproate include fatal hepatic failure, pancreatitis, and severe congenital malformations [2]. Hepatotoxic effects are thought to be related to inhibition of beta-oxidation and resultant mitochondrial toxicity [7,8]. Accordingly, patients with hepatic disease or a family history of mitochondrial disorders are poor candidates for valproate therapy. The incidence of hepatic failure is highest among patients <2 years of age and individuals receiving anticonvulsant polytherapy [8]. The risk of hepatic failure peaks within six months of initiating valproate therapy, irrespective of patient characteristics [7].

## Case presentation

The patient, a 64-year-old male with a past medical history of schizoaffective disorder and dementia, was brought to the hospital by emergency medical services after a fall and altered mental status. On arrival, the patient was found covered in feces and making repetitive noises. The patient was found to be severely bradycardic; cardiology was consulted, and a permanent pacemaker (PPM) was placed on 04/18. He exhibited agitation and received haloperidol 0.5 mg up to three doses a day as needed for two weeks on the medical floor. Psychiatry consultation was initiated, as the patient started to develop symptoms of catatonia, including mutism, stupor, fixed gaze, grimacing, negativism, waxy flexibility, withdrawal, rigidity, and muscle resistance. The patient was transferred to the acute inpatient psychiatric unit for further management. Haloperidol was stopped and the patient was started on 0.5 mg of lorazepam three times a day. The patient showed sustained improvement and eventual resolution of catatonia in the next two weeks. Lorazepam was tapered over the next two weeks without any rebound catatonia but he continue to have intermittent agitation on the unit. A family meeting was held, the medication options were discussed, and the patient’s guardian agreed to start the patient on valproate for underlying schizoaffective disorder, intermittent agitation, and poor sleep.

**Figure 1:**
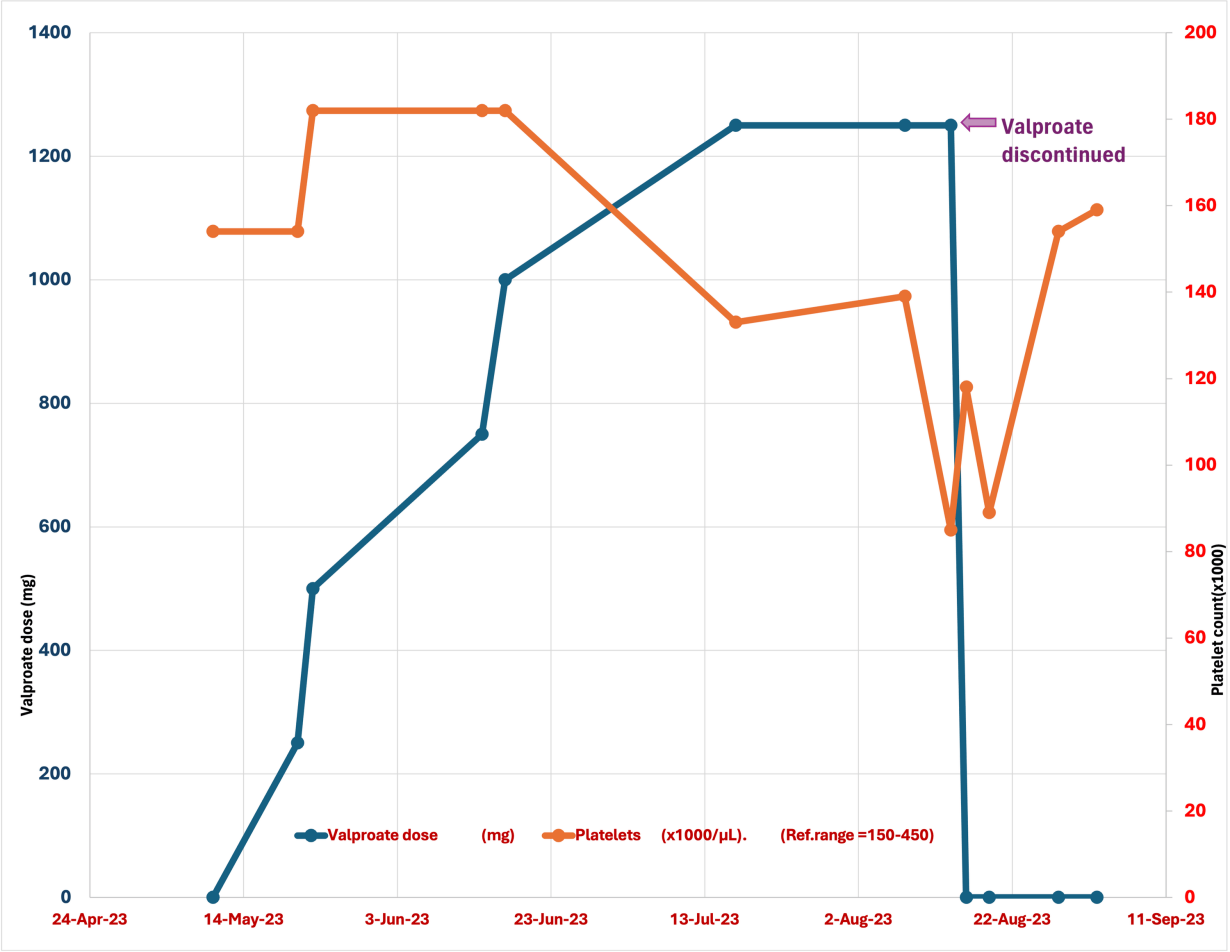
Valproate dosage and corresponding platelet count

The patient was started on 250 mg and slowly titrated to 1250 mg over eight weeks. However, the patient developed thrombocytopenia, suspected to be caused by valproate, leading to its discontinuation. Please refer to Table 1 and Figure 1 for valproate dose titration and respective platelet count. The patient was later switched to risperidone 0.5 mg a day, which worked well, and he was discharged to a nursing facility in stable condition.

**Table 1:** Valproate dosage and corresponding platelet count Platelet count normal reference range=150-450x 1000/μL The table includes valproic levels measured during the titration.

Date	Valproate Dose (mg)	Platelets (x1000/μL)	Valproate Levels (micg/ml)
10-May-23	0	154	
21-May-23	250	154	
23-May-23	500	182	
14-Jun-23	750	182	
17-Jun-23	1000	182	48
17-Jul-23	1250	133	
8-Aug-23	1250	139	70
14-Aug-23	1250	85	
16-Aug-23	0	118	
19-Aug-23	0	89	
28-Aug-23	0	154	
2-Sep-23	0	159	

## Discussion

The risk of thrombocytopenia following the initiation of valproate is a critical consideration, as illustrated by the present case and supported by earlier reports [9]. This risk is especially high during initiation and dose titration, necessitating vigilant monitoring, particularly during these periods [7]. Previous reports have suggested that patients of advanced age, female gender, and those receiving higher doses are more prone to the development of thrombocytopenia [9]. The occurrence of adverse events resulting from valproate-induced thrombocytopenia may be higher in patients taking antiplatelet or anticoagulant medications, both commonly prescribed among geriatric populations. Additionally, patients with lower baseline platelet counts are thought to be at higher risk of developing thrombocytopenia [10].

Thrombocytopenia is a known risk factor for life-threatening events, such as spontaneous bleeding, especially when the levels are very low (i.e. <20 x1000/μL) [11]. The occurrence of these events as a direct result of valproate-induced thrombocytopenia is not well-established [10], indicating the need for further research.

There are no clear guidelines for follow-up labs when using valproate, but clinicians need to use their clinical judgment to check labs within a reasonable timeframe after initiating the medication or during dose titration. Current recommendations include dose reduction or drug discontinuation if hemorrhage, significant bruising, or coagulation abnormalities occur. Weekly complete blood counts should be conducted until platelet levels are within normal range. Since there is potential for thrombocytopenia, it is recommended to obtain platelet counts before starting therapy and periodically thereafter [2].

Several coagulation abnormalities, including thrombocytopenia, platelet function defects, hypofibrogenemia, factor XIII (FXIII) deficiency, and acquired Von Willibrand deficiency, have been linked to the use of valproate. In 2007, a retrospective chart review of 265 patients was conducted to examine the correlation between valproate levels and platelet levels. Within this study, 7.7% of patients experienced at least one episode of thrombocytopenia (platelet count < 100,000) [9]. In previous studies, the frequency of reported valproate-induced thrombocytopenia has ranged from 0%-32%. Researchers have yet to establish a clear mechanism behind valproate-associated thrombocytopenia. Current hypotheses propose immune-mediated destruction of the platelets and direct toxicity to the bone marrow as possible mechanisms. Per package insert, the probability of thrombocytopenia increases significantly at total trough valproate plasma concentrations above 110 mcg/mL in females and 135 mcg/mL in males, though our patient developed at a much lower level [2]. This occurrence of thrombocytopenia at relatively low plasma concentrations underscores the importance of consistent monitoring of platelet levels throughout the initiation and titration of valproate.

It is also important to know drug interactions as co-administration could change the levels and could increase adverse outcomes. Please refer to Tables 2-3 for some clinically important drug interactions.

**Table 2:** Effect of other drugs on valproate level Co-administration of the drugs that increase valproate levels could lead to toxicity if not adjusted. Co-administration of the drugs that decrease valproate levels could make the drug ineffective if not adjusted. Source: Table created by the author from the information in the valproate package insert [2]

Drug Class	Example Drugs	Effect on Valproate Level
Antiseizure drugs	Carbamazepine, Phenytoin, Carbamazepine, and Phenobarbital	Decrease in valproate levels by increasing clearance through enzyme induction
HIV drugs	Ritonavir, Zidovudine	Decrease in valproate levels by increasing clearance through enzyme induction
Certain antibiotics	Ertapenem, Imipenem, Meropenem, Rifampin	Decrease in valproate levels, the mechanism of this interaction is not well understood
Estrogen-containing hormonal contraceptives	Levonorgestrel/Ethinyl estradiol; drospirenone/ethinyl estradiol, drospirenone/estetrol, etc.	Decrease in valproate levels by increasing clearance through enzyme induction
Other drugs	Methotrexate	Decrease in valproate levels
Other drugs	Aspirin	Fourfold increase in valproate levels through the inhibition of metabolism and decreased protein binding
Other drugs	Felbamate	Increase in valproate levels, a mechanism not understood

**Table 3:** Effect of valproate on the levels of other drugs Source: Table created by the author from the information in the valproate package insert [2]

Drug Class	Example Drug	Effect of Valproate on the Levels of Other Drugs
Tricyclic Antidepressants	Amitriptyline, Nortriptyline	Valproate increases levels of Amitriptyline/Nortriptyline
Benzo-diazepam	Diazepam	Valproate increases Diazepam levels by displacing Diazepam from its plasma albumin binding sites and inhibits its metabolism
Antiseizure drugs	Ethosuximide, Lamotrigine, Phenobarbital	Valproate increases levels of Ethosuximide, Phenobarbital, and Lamotrigine. In particular, Lamotrigine levels could be increased by 165%, and Lamotrigine should be reduced when co-administered with valproate to avoid serious skin reactions.

In this patient, the treatment team considered discontinuation before the platelet levels reached a critical low to avoid any possible complications. A rechallenge of valproate and monitoring platelet count would have helped with causality assessment, but we could not try it, as the patient and family do not want to attempt valproate again.

## Conclusions

Even though valproate is effective in treating many neurologic and psychiatric conditions, the medication carries many clinically significant adverse effects. Some can be life-threatening, which further emphasizes the need for close monitoring upon initiating valproate. This case features the temporal relationship between valproate and thrombocytopenia, normal platelet counts prior to medication initiation, decreasing platelet counts during medication administration, and subsequent normalization upon discontinuation of the drug. Despite reports indicating a low incidence of blood coagulation disorders among valproate users, this case demonstrates the imperative need for routine laboratory monitoring in those receiving valproate, given the potential hematologic risks involved.

## References

[1] Torbjörn Tomson, Dina Battino, Emilio Perucca (2016). The remarkable story of valproic acid. Lancet Neurol.

[2] (2024). AbbVie Inc. Depakote package insert. FDA.gov. https://www.accessdata.fda.gov/drugsatfda_docs/label/2024/018723s068lbl.pdf.

[3] (2012). Abbott Labs to pay $1.5 billion to resolve criminal & civil investigations of off-label promotion of Depakote. Company maintained specialized sales force to market drug for off label purposes; targeted elderly dementia patients in nursing homes. https://media.defense.gov/2012/May/07/2001711349/-1/-1/1/abbottPR.pdf.

[4] Owens MJ, Nemeroff CB (2003). Pharmacology of valproate. Psychopharmacol Bull.

[5] Van den Berg RJ, Kok P, Voskuyl RA (1993). Valproate and sodium currents in cultured hippocampal neurons. Exp Brain Res.

[6] Santos R, Linker SB, Stern S (2021). Deficient LEF1 expression is associated with lithium resistance and hyperexcitability in neurons derived from bipolar disorder patients. Mol Psychiatry.

[7] Dreifuss FE, Langer DH (1987). Hepatic considerations in the use of antiepileptic drugs. Epilepsia.

[8] Nanau RM, Neuman MG (2013). Adverse drug reactions induced by valproic acid. Clin Biochem.

[9] Conley EL, Coley KC, Pollock BG, Dapos SV, Maxwell R, Branch RA (2001). Prevalence and risk of thrombocytopenia with valproic acid: experience at a psychiatric teaching hospital. Pharmacotherapy.

[10] Buoli M, Serati M, Botturi A, Altamura AC (2018). The risk of thrombocytopenia during valproic acid therapy: a critical summary of available clinical data. Drugs R D.

[11] Schlappi C, Kulkarni V, Palabindela P, Bemrich-Stolz C, Howard T, Hilliard L, Lebensburger J (2018). Outcomes in mild to moderate isolated thrombocytopenia. Pediatrics.

